# Bifunctional gap-plasmon metasurfaces for visible light: polarization-controlled unidirectional surface plasmon excitation and beam steering at normal incidence

**DOI:** 10.1038/lsa.2017.178

**Published:** 2018-04-20

**Authors:** Fei Ding, Rucha Deshpande, Sergey I Bozhevolnyi

**Affiliations:** 1 SDU Nano Optics, University of Southern Denmark, Campusvej 55, Odense DK-5230, Denmark

**Keywords:** beam steering, bifunctional, gap-plasmon metasurfaces, surface plasmon polaritons

## Abstract

Integration of multiple diversified functionalities into a single, planar and ultra-compact device has become an emerging research area with fascinating possibilities for realization of very dense integration and miniaturization in photonics that requires addressing formidable challenges, particularly for operation in the visible range. Here we design, fabricate and experimentally demonstrate bifunctional gap-plasmon metasurfaces for visible light, allowing for simultaneous polarization-controlled unidirectional surface plasmon polariton (SPP) excitation and beam steering at normal incidence. The designed bifunctional metasurfaces, consisting of anisotropic gap-plasmon resonator arrays, produce two different linear phase gradients along the same direction for respective linear polarizations of incident light, resulting in distinctly different functionalities realized by the same metasurface. The proof-of-concept fabricated metasurfaces exhibit efficient (>25% on average) unidirectional (extinction ratio >20 dB) SPP excitation within the wavelength range of 600–650 nm when illuminated with normally incident light polarized in the direction of the phase gradient. At the same time, broadband (580–700 nm) beam steering (30.6°–37.9°) is realized when normally incident light is polarized perpendicularly to the phase gradient direction. The bifunctional metasurfaces developed in this study can enable advanced research and applications related to other distinct functionalities for photonics integration.

## Introduction

The ability to manipulate light at will is tantalizingly attractive, promising numerous applications. Conventional methods for molding the flow of light typically rely on gradually accumulated phase variations during light propagation, with the resulting devices featuring curved surfaces and complex shapes. These rather bulky configurations do not comply with current trends aiming at very dense integration and miniaturization in photonics. In recent years, optical metasurfaces, that is, optically thin planar arrays of resonant subwavelength elements arranged in a periodic or aperiodic manner, have attracted increasing attention because of their planar profiles and relative ease of fabrication while enabling unprecedented control over optical fields by modifying boundary conditions for impinging optical waves^1, 2, 3, 4^. As such, numerous fascinating applications have been proposed, and promising ultra-compact devices have been accordingly demonstrated by designed metasurfaces, including beam-steering devices^5, 6, 7, 8, 9, 10^, surface waves or waveguide couplers^11, 12, 13, 14, 15, 16, 17, 18^, focusing lenses^19, 20, 21, 22, 23^, optical holograms^24, 25, 26, 27, 28, 29^, coding metasurfaces^30, 31, 32^, waveplates^33, 34, 35, 36^ and polarimeters^37, 38, 39, 40, 41^.

However, most state-of-the-art metasurfaces are designed for a single functionality or identical/similar functionalities (for example, polarization-dependent beam steering^9, 10^, surface waves coupling^13, 14, 15^ and holograms^24, 27, 28, 29^), not quite reaching the desired goal of realizing distinctly different functionalities. Metasurfaces that facilitate efficient integration of multiple diversified functionalities into a single ultrathin device with a compact footprint have become an emerging research area. Recently, Hasman and colleagues proposed a generic approach to realizing multifunctional metasurfaces via the synthesis of shared-aperture antenna arrays and geometric phase concepts^42^. By randomly mixing optical nanoantenna subarrays, where each subarray provides a different phase function in a spin-dependent manner, multiple wave fronts with different functionalities can be achieved within a single shared aperture^40, 42, 43^. However, the implemented approach suffers from intrinsic crosstalk between different subarrays, and the efficiency of each functionality is inevitably limited. To design multifunctional metasurfaces for linear polarization, particularly bifunctional devices, metasurfaces composed of anisotropic meta-atoms with polarization-sensitive phase responses have been found to be promising for realizing distinct functionalities with very high efficiencies and low crosstalk^44^. While bifunctional metasurfaces have been successfully demonstrated in the microwave range^44^, metasurfaces processing multiple distinct functionalities at visible wavelengths still remain largely unexplored.

In this paper, we design, fabricate and experimentally demonstrate bifunctional gap-plasmon metasurfaces for visible-light operation that enable simultaneous polarization-controlled unidirectional surface plasmon polariton (SPP) excitation and beam steering at normal incidence. The bifunctional metasurfaces, consisting of anisotropic gap-plasmon resonator arrays, produce two different linear phase gradients along the same direction for the respective linear polarizations of incident light, resulting in distinct functionalities realized with the same metasurface. The proof-of-concept fabricated metasurfaces exhibit efficient (>25% on average) unidirectional (extinction ratio >20 dB) SPP excitation within the wavelength range of 600–650 nm when illuminated with normally incident light polarized in the direction of the phase gradient. At the same time, broadband (580–700 nm) beam steering (30.6°–37.9°) is realized when normally incident light is polarized perpendicularly to the phase gradient direction.

## Materials and methods

### Simulation

**Figure 1:**
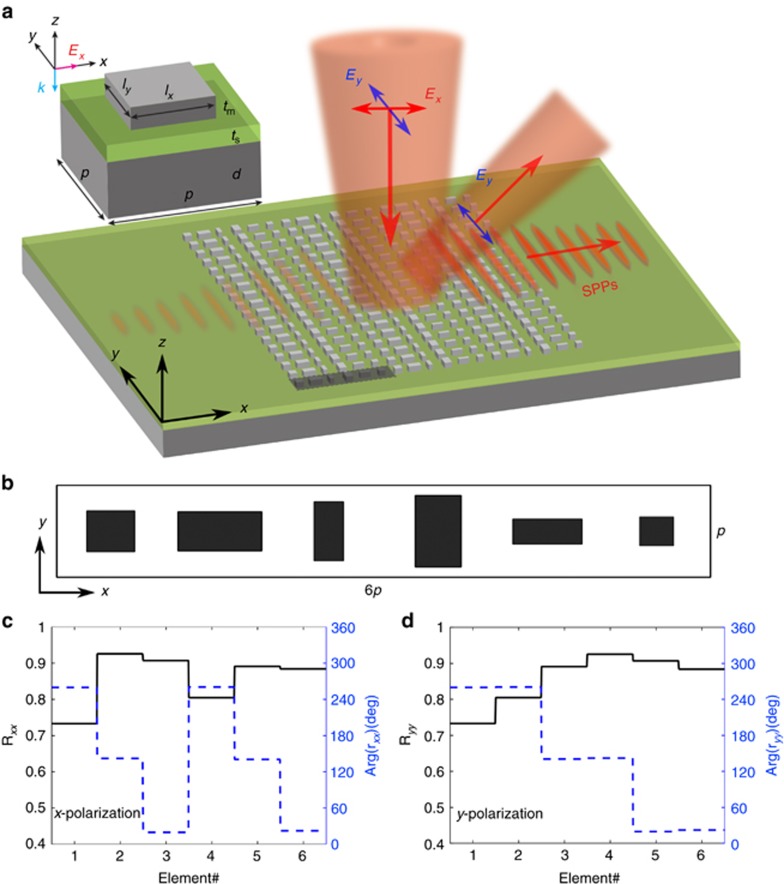
Working principle of the bifunctional gap-plasmon metasurface for visible light. (**a**) Artistic rendering of the working principle: different polarization components are selectively coupled into SPPs (*x*-polarization) or anomalously reflected (*y*-polarization). The gray region represents the supercell composed of six unit cells. The top panel shows the schematic of the unit cell consisting of an Ag nanobrick on top of a spacer and Ag substrate. The fixed geometrical parameters are *p*=190 nm, *d*=150 nm, *t*_s_=35 nm, and *t*_m_=40 nm. (**b**) Top view of the metasurface supercell composed of six nanobricks. (**c** and **d**) The corresponding reflection amplitudes and phases of the associated six nanobricks at *λ*=633 nm for *x*-polarization (**c**) and *y*-polarization (**d**).

**Figure 2:**
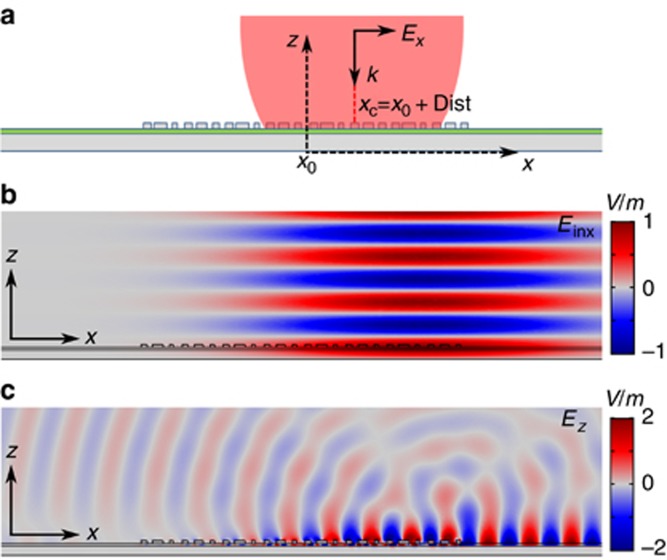
Simulated performance of the unidirectional SPP excitation for *x*-polarization at *λ*=633 nm. (**a**) Side view of the SPP coupler composed of four supercells along the *x*-axis. An *x*-polarized Gaussian beam is propagating normal to the surface. (**b**) The electric field of the incident *x*-polarized Gaussian beam (*w*_0_=2 μm). The offset of Gaussian beam from the center of the SPP coupler is Dist=*x*_c_−*x*_0_=1.71 μm. (**c**) The *z*-component of the electric field, corresponding to the transverse electric field component of SPPs.

**Figure 3:**
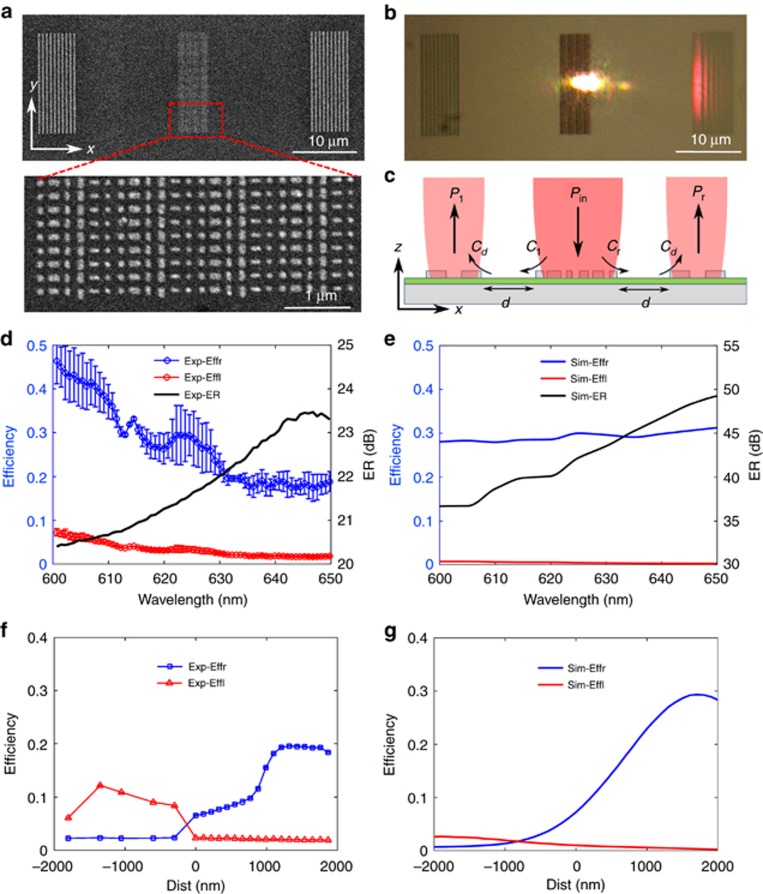
Optical characterization of the SPP coupling for *x*-polarization. (**a**) SEM image of the SPP coupling device (scale bar 10 μm) and part of the SPP coupler (scale bar 1 μm). (**b**) Optical image of the SPP excitation with a broadband excitation source where the right-coupling efficiency *C*_r_ is maximized (scale bar 10 μm). (**c**) Schematic of the sample layout for deriving the coupling efficiencies incorporating the SPP coupler and two identical decoupling gratings. (**d** and **e**) Measured (**d**) and simulated (**e**) coupling efficiencies and extinction ratio with an optimally positioned incident laser beam. (**f** and **g**) Measured (**f**) and simulated (**g**) SPP excitation efficiencies versus the position of a scanned laser beam at the design wavelength of *λ*=633 nm. Exp-Effl, Experimental Efficiency to left side; Exp-Effr, Experimental Efficiency to right side; Exp-ER, Experimental ER; Sim-Effl, Simulated Efficiency to left side; Sim-Effr, Simulated Efficiency to right side; Sim-ER, Simulated ER.

**Figure 4:**
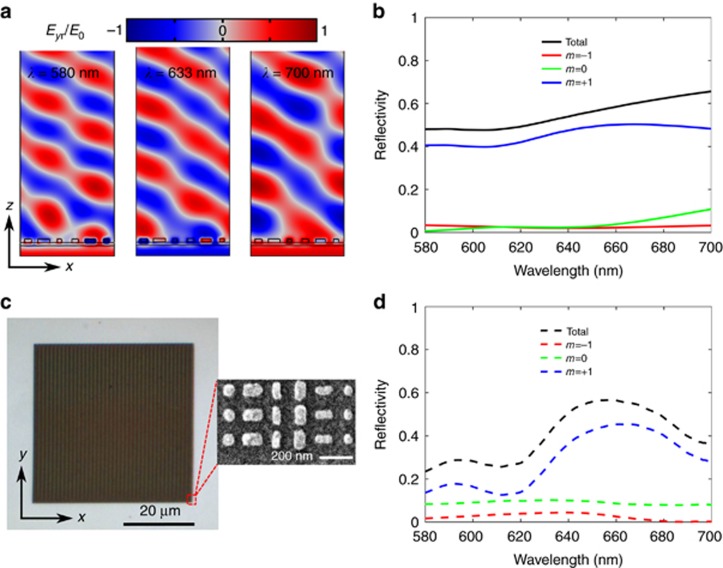
Simulation and characterization of the broadband beam steering for *y*-polarization. (**a**) Electric field *E*_*y*r_ distributions at 580, 633 and 700 nm. The *y*-polarized plane wave is normally incident on the metasurface. (**b**) Calculated diffraction efficiencies of orders 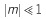 for *y*-polarization. (**c**) Optical image of the fabricated sample (scale bar 20 μm), and SEM image of part of the fabricated sample (scale bar 200 nm). (**d**) Measured diffraction efficiencies for orders 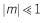 for *y*-polarization.

All three-dimensional (3D) simulations were performed using the commercially available software Comsol Multiphysics (ver. 5.2) based on the finite element method (FEM). For the periodic homogeneous gap-plasmon metasurfaces (Figure 1a), we modeled one unit cell by applying periodic boundary conditions on the vertical sides of the cell. The complex reflection coefficients were determined with respect to the nanobrick top surfaces with linearly polarized light normally incident on the metasurface. The permittivity of silver (Ag) was described by the Drude model fitted with experimental data^45^, and the damping constant *ω*_d_ was increased by a factor of three to consider the additional losses caused by surface scattering and grain boundary effects in thin films (Supplementary Information Section S1). The silicon dioxide (SiO_2_) spacer layer was considered a lossless dielectric with a constant refractive index *n*=1.45. The medium above the metasurface was chosen to be air and truncated using the perfectly matched layer (PML) to minimize reflection. For an SPP coupler consisting of four supercells in the *x*-direction and infinitely extended in the *y*-direction (Figures 2 and 3), the *x*-polarized Gaussian input beam was considered invariant along the *y*-direction, and PMLs were used in the *x*- and *z*-directions. The SPP power was obtained by integrating the *x*-component of Poynting’s vector on a vertical plane 10 μm away from the SPP coupler. Then, we calculated the corresponding coupling efficiency and extinction ratio. Note that the coupling efficiency was corrected for the exponential damping of the excited SPPs over the propagation distance between the coupler and the evaluation plane. Regarding beam steering (Figure 4a), the *y*-polarized plane wave was considered to be normally incident on the metasurface supercell with periodic boundary conditions set in both the *x*- and *y*-directions.

### Fabrication

All investigated samples were fabricated using standard thin-film deposition and electron-beam lithography (EBL) techniques. First, successive layers of 3 nm Ti, 150 nm Ag, 3 nm Ti and 35 nm SiO_2_ were deposited onto a silicon substrate using electron-beam evaporation (Ti and Ag) and RF-sputtering (SiO_2_). Then, the metasurface was defined using EBL employing a 100-nm-thick PMMA (2% in anisole, Micro Chem) layer at an acceleration voltage of 30 keV. After development in a 1:3 solution of methyl isobutyl ketone (MIBK) and isopropyl alcohol (IPA), a 3-nm Ti adhesion layer and a 40-nm Ag layer were deposited subsequently using electron-beam evaporation. The Ag patterns were finally formed on top of the SiO_2_ film after a lift-off process.

### Optical characterization

The performance of SPP couplers was studied using a homemade spatially resolved linear reflectance spectroscopy device featuring an IX71 microscope (Olympus) equipped with a broadband supercontinuum white light source (NKT), spectral filters, polarizers, CCD, and a fiber-coupled grating spectrometer QE65000 (Ocean Optics). The light from the two decoupling gratings was collected in the backscattering configuration using an MPlanFL (Olympus) objective with 100 × magnification (numerical aperture (NA)=0.9). The image area analyzed by the spectrometer was limited by a homemade pinhole, resulting in a circular probing area with a diameter of ~15 μm. By positioning the pinhole, the light from the right- and left-side gratings could be selectively chosen. Prior to measurements, the incident Gaussian beam was focused onto a 35-nm-thick SiO_2_-coated Ag substrate to check the beam waist and the incident power. The incident power on the SPP coupler was determined using the formula *P*_in_=*P*_R_/*R*, where *P*_R_ is the reflected power and *R* is the reflectivity of the planar SiO_2_–Ag film. After normalizing the collected light from the decoupling gratings by the incident power, the total coupling efficiency of the three-component device was finally determined.

## Results and discussion

The working principle of the proposed visible bifunctional gap-plasmon metasurface, which is simultaneously capable of efficiently and unidirectionally exciting SPPs under the *x*-polarized illumination and anomalously steering the reflected *y*-polarized light, is schematically illustrated in Figure 1. In contrast to previously reported polarization-controlled metasurfaces processing two orthogonal phase gradients upon reflection for orthogonal linear polarizations^15^, here two different linear phase gradients, *ζ*_*x*_ and *ζ*_*y*_, are introduced along the same direction (that is, the *x*-direction) for respective orthogonal linear polarizations of incident light, resulting in two different functionalities in the *x*–*z* plane. Specifically, the reflection phase gradient for *x*-polarization is equal to the wave vector of SPPs propagating along the air-dielectric-metal interfaces (that is, *ζ*_*x*_=*k*_SPP_), while the magnitude of the phase gradient is smaller than the light wavenumber in free space when the incident light is *y*-polarized (that is, *ζ*_y_<*k*_0_). Thus, the metasurface can simultaneously convert the *x*-polarized normally incident light into SPPs propagating along the *x*-axis (in the direction determined by the sign of the phase gradient) and anomalously reflect the *y*-polarized (normally incident) light at an oblique angle.

To design the gap-plasmon-based phase gradient metasurface, we first consider the metal–insulator–metal configuration without phase gradients. Similar to the design procedure of our previous work on polarization-controlled SPP excitation^15^, the period *p* of the unit cell is predominantly determined by the corresponding SPP wavelength *λ*_SPP_ and the number of discretized phase steps *N*, namely, *p*=*λ*_SPP_/*N*. For normally incident light at the design wavelength of *λ*=633 nm and a 35-nm-thick SiO_2_ film on top of a thick Ag layer, the SPP wavelength is estimated to be ~570 nm (Supplementary Information Section S2). To avoid nanometer-sized dimensions and thereby relax the fabrication requirements, we discretize the 2*π* phase range into three equal steps and select six elements with a center-to-center distance of *p*=190 nm to create a supercell to be periodically repeated in the *x*- and *y*-directions (Figure 1a and 1b). By tailoring the dimensions (*l*_*x*_ and *l*_*y*_) of the top Ag nanobrick, we can independently control the amplitude and phase of reflected light for a homogeneous (that is, without gradients) metasurface at the design wavelength of *λ*=633 nm for two orthogonal linear polarizations (Supplementary Information Section S3). In Figure 1c, the solid black and dashed blue curves correspond, respectively, to the co-polarized reflectivity and reflection phase for the six selected nanobricks and the *x*-polarized incident light. The supercell composed of six nanobricks provides a 4*π* phase span with a constant phase shift of 2*π*/3 between the neighboring elements, resulting in unidirectional SPP excitation because of the phase gradient *ζ*_*x*_ that compensates for the momentum mismatch between the propagating light and SPPs^11, 15^. At the same time, the supercell provides only a 2*π* phase span for *y*-polarization, which is achieved by maintaining a similar *y*-dimension for two neighboring nanobricks (that is, for nanobricks 1 and 2, 3 and 4, and 5 and 6, as shown in Figure 1b) that thereby provide the same phase in reflection (Figure 1d). Therefore, the incident *y*-polarized light at a wavelength of 633 nm will anomalously be reflected at an angle of 33.7° in the *x*–*z* plane according to the generalized Snell’s law^5^. It should be noted that we disregard variations in reflection amplitudes produced by different elements comprising the supercell, which could slightly affect the performance. In addition, although the linear phase gradient is designed for a nominal wavelength of 633 nm, the gradient exhibits only a weak wavelength dependence, thus allowing for broadband unidirectional SPP excitation and beam steering.

In the following, we first numerically evaluate the performance of the designed bifunctional metasurface as an SPP coupler for *x*-polarization. The considered metasurface consists of four supercells in the *x*-direction, corresponding to an overall lateral dimensional of *L*_c_=24*p*=4.56 μm, and is assumed to extend infinitely in the *y*-direction (the side view is shown in Figure 2a). In the simulations, an *x*-polarized Gaussian beam with the waist *w*_0_ equal to 2 μm propagates normal to the surface (see Materials and Methods section for details). It is noted that the number of supercells constituting the SPP coupler can be varied, and in principle, both this number and the incident beam size should be adjusted coherently to maximize the SPP coupling efficiency. To maximize the excitation efficiency of SPPs in the +*x*-direction, the incident Gaussian beam is displaced with respect to the coupler center in the direction of the desired SPP propagation (Figure 2b). The calculated distribution of the normal to the surface electric field component (Figure 2c) clearly indicates that the SPPs are predominantly excited and routed into the +*x*-direction while the unwanted SPPs propagating in the opposite direction are strongly suppressed. The well-pronounced unidirectional SPP excitation is ascribed to the realization of the appropriate quasi-linear reflection phase gradient as well as to the off-center positioning of the incident beam, which reduces the excited SPP attenuation inside the SPP coupler while further suppressing the oppositely propagating SPPs. It should be emphasized that, while the maximum unidirectionality in the SPP excitation (in the +*x*-direction) is achieved by positioning the incident beam away from the coupler center, the SPP coupler does exhibit intrinsic unidirectional SPP excitation when the Gaussian beam is normally incident at the center of the SPP coupler (Supplementary Information Section S4). To quantitatively describe the performance of the SPP coupler, we integrate the power of excited SPP signals propagating in the +*x*- and −*x*-directions (see Materials and Methods section for details). After normalization, the coupling efficiency *C*_r_ (*C*_l_) is determined; *C*_r_≅29.4% (*C*_l_≅0.34%) in our case. The corresponding extinction ratio (ER) between the right- and left-propagating SPPs, defined as ER=10 × ln (*C*_r_/*C*_l_)^46^, is found to be ~44.4 dB, which is in excellent agreement with our visual observation (Figure 2c).

Finally, it is worth noting that the performance of the SPP coupler is inherently limited by the propagation attenuation as well as by the scattering due to intra- and intersupercell discontinuities^12^. For example, the generated SPPs are partially absorbed by the gap-plasmon elements and scattered out because of surface discontinuity when propagating along the metasurface. Additionally, the SPP wavelength in the coupler region is slightly smaller than the outside air-SiO_2_–Ag interface region, implying a non-optimal phase-matching condition for the chosen supercell period. Furthermore, the calculated coupling performance depends considerably on the permittivity of the metal^47^. Taking all the above mentioned factors into account, it becomes clear that the performance of the SPP coupler can further be improved by iteratively optimizing the supercell period and geometrical parameters of the nanobricks^15^, as well as by using epitaxial Ag with intrinsically lower loss to construct the top nanobricks^48^.

To experimentally validate the functionality of unidirectional SPP excitation, a sample was fabricated using standard electron-beam lithography (EBL) and a lift-off process (see more details about the fabrication process in the Materials and Methods section). Figure 3a displays the scanning electron microscopy (SEM) image of the whole device for SPP characterization, which consists of the central SPP coupler (MSS1) and two identical decoupling gratings on the right and left sides for coupling the SPPs into free-space photons. The SPP coupler has a lateral dimension of 4.56 μm (4 supercells along the *x*-direction), and its length is ~15 μm. Each decoupling grating features ten ridges, with the period and width equal to 570 and 285 nm, respectively. The distance between the central SPP coupler and the decoupling gratings is *d*=15 μm, which is sufficient to eliminate scattering within the coupler region.

Following fabrication, we characterized the SPP excitation of MSS1 using a homemade spatially resolved linear reflection spectroscopy device that can selectively capture the decoupling light from the gratings on both sides (see Materials and Methods section for more details). Using an *x*-polarized Gaussian beam with the waist estimated to be ~2 μm and optimally positioned to maximize the coupling efficiency of the right-propagating SPPs, Figure 3b shows the corresponding CCD image with a broadband excitation source, verifying the unidirectional excitation of SPPs over a wide spectrum. Strongly scattered light is observed only from the decoupling grating sitting on the right side, whereas practically no light is coupled out and observed from the left grating, implying that the right-propagating SPPs are dominant while the left ones are strongly suppressed. In contrast, no light is coupled out and observed from the two gratings when the incident light is *y*-polarized; validating that SPP excitation only exists with *x*-polarized excitation (Supplementary Information Section S5).

By normalizing the collected power of the scattered light from the two-sided gratings to the incident power, we can determine the total coupling efficiencies of the whole device composed of the SPP coupler and gratings, namely, *C*_tot_=*P*/*P*_in_, where *P* is the power collected from the grating and *P*_in_ is the incident power. Given the coupling of the SPP coupler, the damping of launched SPPs over the distance *d*, and the decoupling of gratings, we can derive the coupling efficiencies of the central SPP coupler. As sketched in Figure 3c, the total coupling efficiencies of the three-component device can be expressed as *C*_tot_=*C* × exp(−*d*/*L_p_*) × *C*_de_=*P*/*P*_in_, where *L_p_* is the propagation length of the SPPs supported on the air-SiO_2_-Ag interface and *C*_de_ is the decoupling efficiency of the gratings. To calculate the coupling efficiency *C*, we first measured the propagation length *L_p_* using the established method^4^; the values closely matched the calculated SPP propagation length (Supplementary Information Section S6). Hereafter, we assume that the experimental value of the decoupling efficiency of the gratings *C*_de_ is equal to the theoretical value. Numerical simulations showed that the non-uniform decoupling grating profiles degrade the decoupling efficiencies *C*_de_. Thus, we can expect that the experimental decoupling efficiency *C*_de_ is less than its computed value, for example, 78.5% at *λ*=633 nm (Supplementary Information Section S7). As such, we can reasonably obtain the lower bound of the coupling efficiency *C* (Ref. 49). On the basis of this assumption, the measured maximum launching efficiency for the right-propagating SPPs *C*_r_ is found to be at least equal to ~19.6% at the design wavelength of *λ*=633 nm, which is slightly lower than the theoretically predicted value of ~29.4%. Figure 3d shows the measured coupling efficiencies over the wavelength range from 600 to 650 nm, where an averaged coupling efficiency *C*_r_ of >25% has been achieved, superior to values reported for a metasurface-based SPP coupler at visible and near-infrared wavelengths^13, 14^. Compared with the slightly varied simulation results (Figure 3e), the experimental coupling efficiencies are more sensitive to the incident wavelength. In addition to the hypothesis regarding the coupling efficiencies, we believe the discrepancy is related to imperfections and the surface roughness of the fabricated nanobricks, different excitation conditions, and the uncertainty in the material properties of the evaporated Ag film as well as the increased damping related to the Ti adhesion layer between the Ag and SiO_2_ layers. To further characterize the unidirectional coupling properties, we extracted the corresponding ER as a function of incident wavelength. As shown in Figure 3d (black curve), the measured ER is greater than 20 dB between 600 and 650 nm. Moreover, the measured ER is in qualitative agreement with the calculated ones, as shown in Figure 3e (black curve). However, because of the increased unwanted SPP signal propagating to the left side in the experiment, the measured ER is ~15 dB lower than the calculated value.

As previously mentioned, the coupling efficiency *C*_r_ is sensitive to the position of the excitation beam. To gain more insight into the position dependence, we scanned the laser beam across the SPP coupler and evaluated the power carried with the SPPs propagating in opposite directions (Figure 3f). When the beam is well positioned in the center, the measured coupling efficiencies of the SPPs propagating to the right and left are, respectively, ~6.6% and ~2.1%, indicating that the unidirectional SPP excitation is in good agreement with the simulations predicting *C*_r_=7.2% and *C*_l_=1%, respectively (Supplementary Information Section S4). While the beam is scanned from the center toward the increasing coordinate (that is, Dist >0), the left excitation efficiency *C*_l_ decreases monotonically. In contrast, the coupling efficiency *C*_r_ of the right-propagating SPPs increases first, reaching the maximum at the position with the offset Dist ≅1430 nm, and then decreases when the beam moves outward, which is attributed to the fact that the generated SPPs always suffer from non-negligible scattering caused by intra- and inter-supercell discontinuities. When the beam moves further and nears the right edge of the SPP coupler, although the generated right-propagating SPPs experience less scattering loss, the interaction between the incident light and SPP coupler becomes weaker, as only part of the structure is effectively illuminated by the incident light; thus, the value of *C*_r_ is reduced. If the laser beam moves to the left side from the center (that is, Dist<0), the left excitation efficiency *C*_l_ increases first and then decreases, while the right coupling efficiency *C*_r_ decreases monotonically, approaching 0. Compared with the simulated position-dependent SPP excitation (Figure 3g), there is a reasonable agreement between the experimental and simulated position dependencies with respect to their shape; however, the optimal position is slightly shifted, and the simulated SPP excitation is more unidirectional than that observed in the experiment, ensuring ~85 times more efficient SPP excitation to the right than to the left. There is also a discrepancy in the absolute value of the coupling efficiency. These differences can be explained by the inaccuracy of the laser beam position, together with the aforementioned imperfections of the fabricated nanobricks, different excitation conditions, and the uncertainty in the material properties. More experimental results pertaining to the position-dependent coupling efficiencies at other wavelengths are presented in Supplementary Information Section S8, validating the broadband unidirectional SPP excitation. As a final comment, it should be emphasized that the coupling efficiencies *C*_r_ and *C*_l_ show asymmetrical position dependencies when the laser beam is scanned from the coupler center to the edges, which are distinctly different from the symmetrical position-dependent SPP excitation induced by a regular grating (Supplementary Information Section S9)^50^. This drastic difference is ascribed to the unidirectional phase gradient in reflection by the designed metasurface.

In addition to supporting unidirectional SPP excitation under *x*-polarization, our metasurface can function as a broadband beam steerer for *y*-polarized light. In the following, we discuss the functionality of beam steering for *y*-polarization. For a supercell with a periodicity of 1140 nm, a 2*π* phase span is introduced for *y*-polarized light along the *x*-direction at *λ*=633 nm, resulting in an anomalous reflection peak at an angle of 33.7° in the *x*–*z* plane. To verify the broadband beam steering, 3D full-wave numerical simulations were first performed by modeling the periodic supercell shown in Figure 1b (see Materials and Methods section). The reflected electric fields *E*_*y*r_ at several wavelengths (580, 633 and 700 nm, respectively) are plotted in Figure 4a, showing well-defined wave fronts. As expected, ~90% of the reflected light is contained within the +1 diffraction order at the design wavelength of *λ*=633 nm, while the other diffraction orders are strongly suppressed (Figure 4b). Therefore, the reflected beam at *λ*=633 nm has less distortion, and the wave front closely resembles an ideal plane wave. When the working wavelength deviates further from the designed value, for instance, *λ*=700 nm, the unwanted diffraction orders increase, resulting in a more undulatory and inhomogeneous wave front. Although there is a slight disturbance, the broadband light steering is sustained over the wide spectral range of 580–700 nm, and the corresponding steering angle is varied from 30.6° to 37.9°, respectively. In contrast to the case of *y*-polarization, there is practically no anomalous reflection, and nearly all the reflected light goes to zero order once the incident polarization is switched to *x*-polarization (Supplementary Information Section S10), in accord with the previous part of the SPP excitation. As a final comment, we note that the total reflectivity is limited because of ohmic losses in the metals.

To experimentally investigate the beam-steering property, we fabricated another sample (MSS2) following the same procedure as before (see Materials and Methods section). The overall lateral size of MSS2 was approximately 45 × 45 μm^2^, comprising 40 × 240 supercells (Figure 4c). In principle, we could use MSS1 shown in Figure 3 to characterize the beam steering for *y*-polarization. However, in our homemade experimental setup for diffraction characterization^51^, the beam spot size was ~15 μm, which exceeds the MSS1 area. Illuminating MMS2 by a *y*-polarized wave at normal incidence, we measured the zero- and first-order diffraction efficiencies, as shown in Figure 4d. In general, reasonable agreement is observed between the measured and calculated diffraction efficiencies, verifying the broadband steering for *y*-polarized light, albeit with some discrepancies regarding the efficiencies, particularly at short wavelengths. Specifically, the measured efficiencies of the total reflection and +1 order diffraction are reduced by ~10% compared with the calculated values near the design wavelength, and the unwanted zero-order diffraction is not completely suppressed over the investigated wavelength range, which we ascribe to the imperfections of the fabricated nanobricks (the SEM image of Figure 4c), together with the uncertainty in the practical optical constants of the evaporated Ag film and Ti adhesion layer.

## Conclusion

In this work, we have proposed and demonstrated bifunctional gap-plasmon metasurfaces for operation at visible wavelengths. The metasurfaces consist of anisotropic gap-plasmon resonator arrays providing two different linear phase gradients along the same direction for respective linear polarizations of incident light, thereby allowing for simultaneous polarization-controlled unidirectional SPP excitation and beam steering at normal incidence. The proof-of-concept fabricated metasurfaces exhibit efficient unidirectional SPP excitation over the wavelength range of 600–650 nm with an average coupling efficiency of >25% and extinction ratio exceeding 20 dB under normal illumination with an *x*-polarized beam. Moreover, broadband (600–650 nm) beam steering has been experimentally realized for *y*-polarization. Although the aforementioned functionalities are demonstrated at normal incidence, it should be noted that the designed metasurface can also operate rather well at oblique incidence (Supplementary Information Sections S11 and S12). Finally, it should be noted that higher degrees of functionality, for example, three different functions, can be realized by designing more complex unit cells or using segmented or interleaved configurations^40, 41^. Owing to compactness and integration compatibility, we believe that the proposed visible-wavelength bifunctional metasurfaces promise high performance, low crosstalk, and polarization-controlled distinct functionalities for more advanced applications related to integrated hybrid plasmonic and photonics circuits^52, 53^.
