# Supplementary material for: Bifunctional gap-plasmon metasurfaces for visible light: polarization-controlled unidirectional surface plasmon excitation and beam steering at normal incidence
Source: Light Sci Appl. 2018 Apr 20;7:17178–. doi: 10.1038/lsa.2017.178 (PMC6060058; doi:10.1038/lsa.2017.178)
Supplement: Supplementary Material [file lsa2017178x1.docx]

Supplementary Information

Bifunctional Gap-Plasmon Metasurfaces for Visible Light: Polarization-Controlled Unidirectional Surface Plasmon Excitation and Beam Steering at Normal Incidence

Fei Ding*, Rucha Deshpande, and Sergey I. Bozhevolnyi

SDU Nano Optics, University of Southern Denmark, Campusvej 55, DK-5230 Odense, Denmark

*e-mail: [feid@mci.sdu.dk](mailto:feid@mci.sdu.dk)

**Section S1: Optical constant of Ag**

The permittivity of Ag was described by the Drude model fitted with experimental data,^1^

$$\varepsilon\left( \omega\right)=\varepsilon_{\infty}-\frac{\omega_{p}^{2}}{\omega^{2}+i\alpha\omega_{d}\omega}$$

where *ε*_∞_ = 3.4, *ω*_p_ = 1.3823 × 10^16^ s^-1^, *ω*_d_ = 3 × 10^13^ s^-1^, *α* is a loss factor considering the additional loss caused by the surface scattering and grain boundary effects of the fabricated thin films. In all the simulations, *α* was set to be 3. It should be noted that similar procedures have been used in other studies (see, for example, Ref. 2).

**Section S2: Effective mode index of the SPPs bounded on the air-SiO_2_-Ag interface**


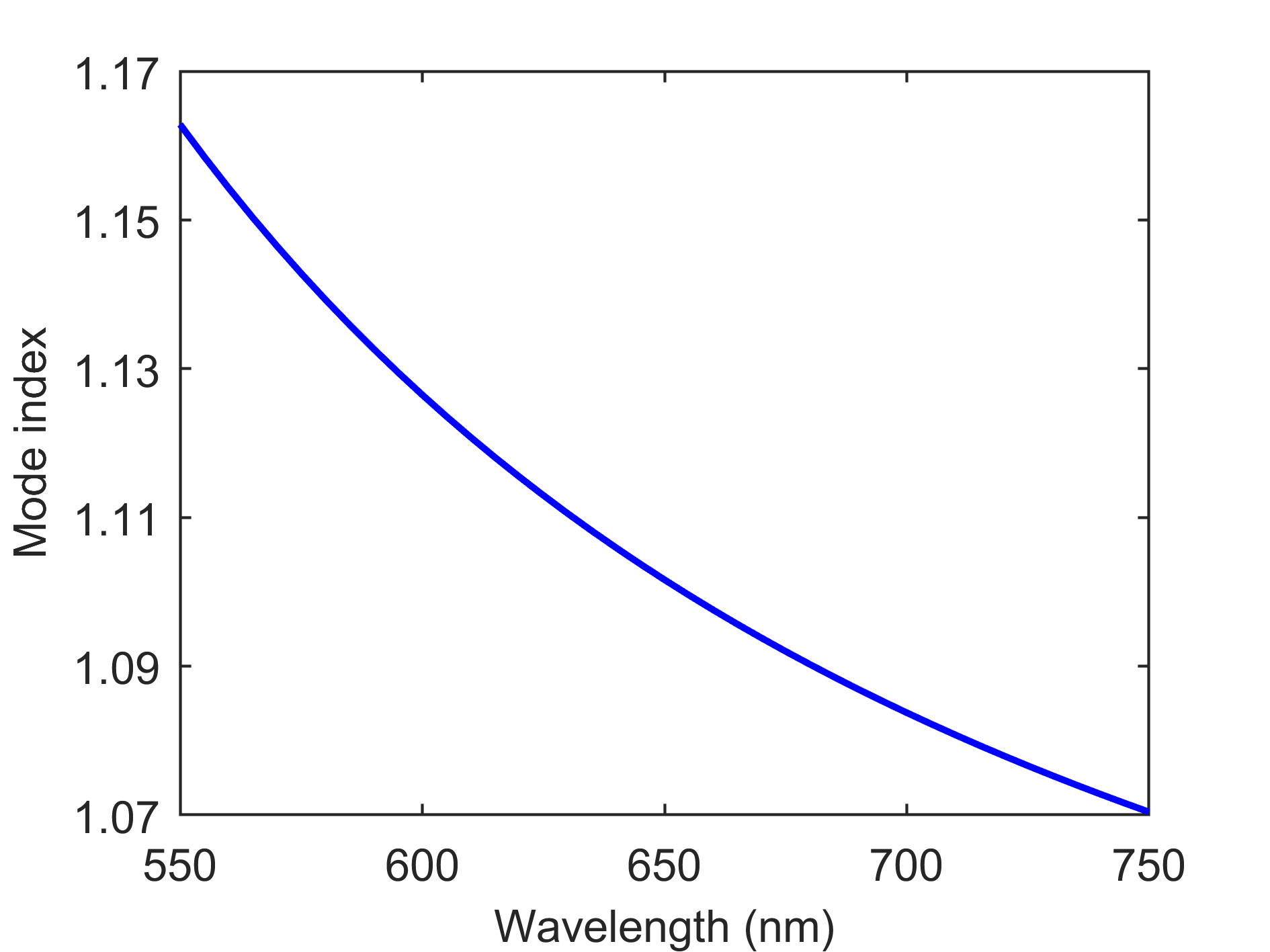


**Figure S1** Calculated effective mode index of the interface consisting of a 35-nm-thick SiO_2_ film on top of the Ag substrate.

**Section S3: Calculated reflection amplitude and phase as a function of nanobrick dimensions at *λ* = 633 nm.**

**
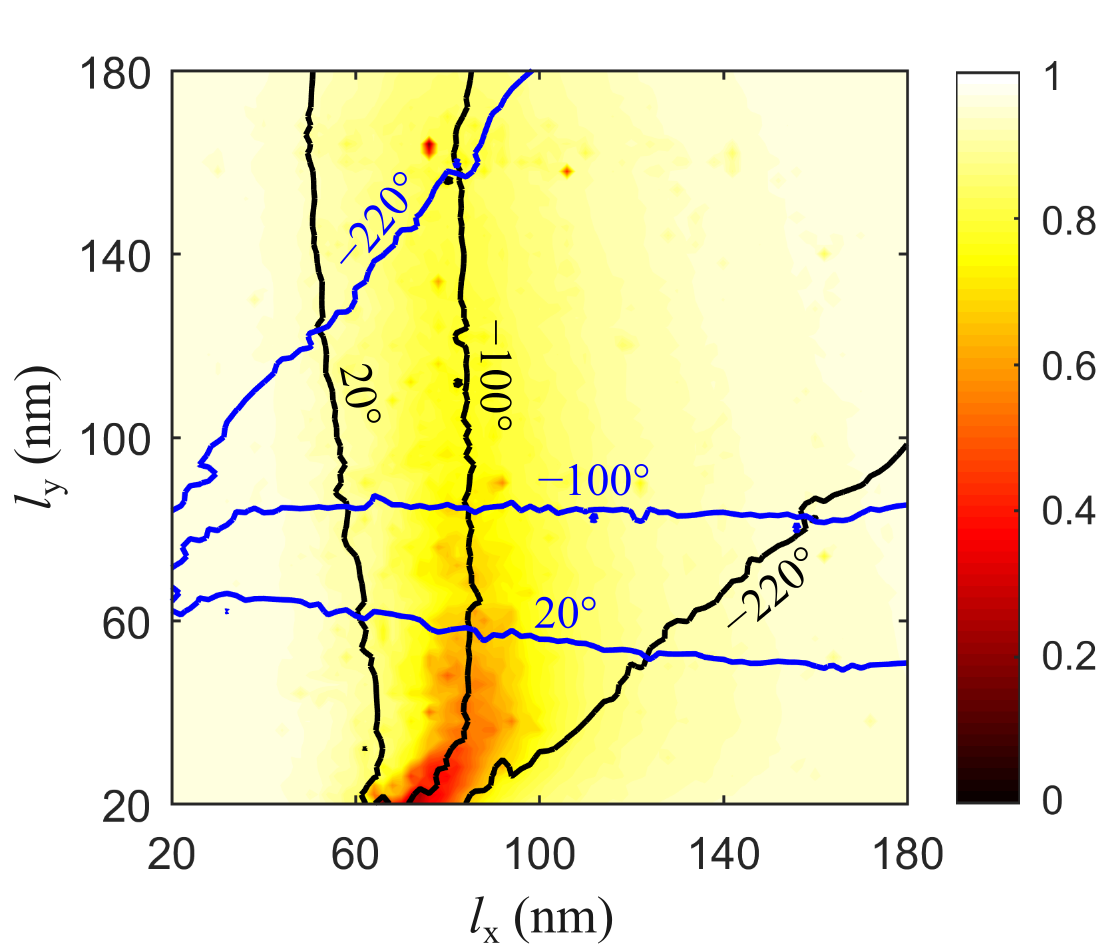
**

**Figure S2** Calculated reflectivity as a function of nanobrick dimensions for *λ* = 633 nm. Reflectivity map for *x*-polarization, while lines are contours of the reflection phase for both *x*- and *y*-polarization. Note that the reflectivity for *y*-polarization can be obtained by mirroring the map for *x*-polarization along the line *l*_x_ = *l*_y_.

**Section S4: Simulated unidirectional SPP excitation for *x*-polarization at *λ* = 633 nm when the Gaussian beam is propagating normal to the center of the coupler**

**
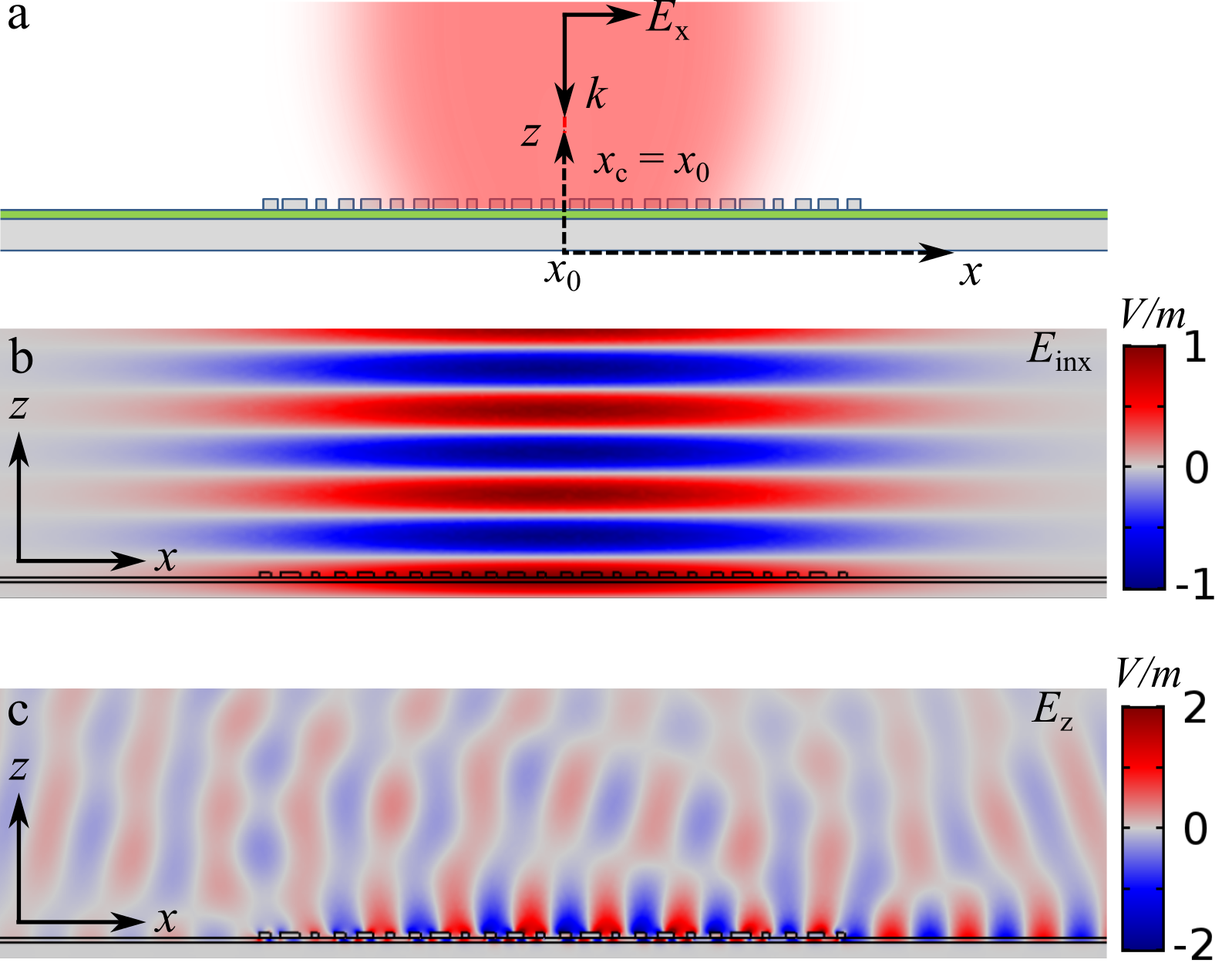
**

**Figure S3** Simulated unidirectional SPP excitation for *x*-polarization at *λ* = 633 nm when the Gaussian beam is propagating normal to the center of the coupler. (**a**) Side view of the SPP coupler composed of four supercells along the *x*-axis. (**b**) The electric field of the incident *x*-polarized Gaussian beam (*w*_0_ = 2 μm). (**c**) The *z*-component of the electric field, corresponding to the transverse electric field component of SPPs. The coupling efficiency *C*_r_ (*C*_l_) is estimated to be *C*_r_ ≅ 7.2% (*C*_l_ ≅ 1%), indicating intrinsic unidirectionality in the SPP excitation.

**Section S5: Optical image of the device excited with a *y*-polarized broadband source**

**
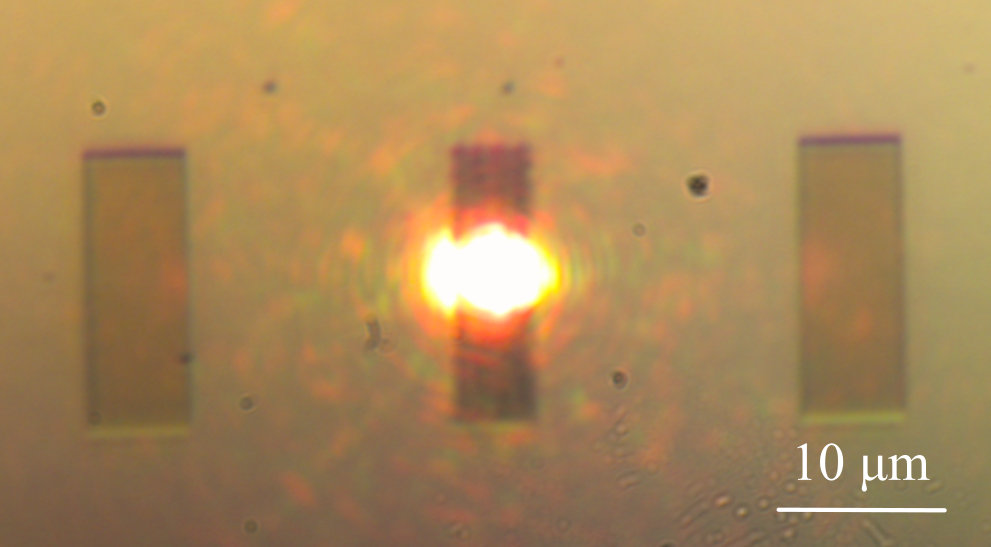
**

**Figure S4** Optical image of the device excited with a *y*-polarized broadband source. No light is coupled into SPPs and finally decoupled out from the two gratings. The intensity of incident light is greatly enhanced to make sure that no SPPs coupling exists.

**Section S6: Propagation length of the SPPs propagating along on the air-SiO_2_-Ag interface**


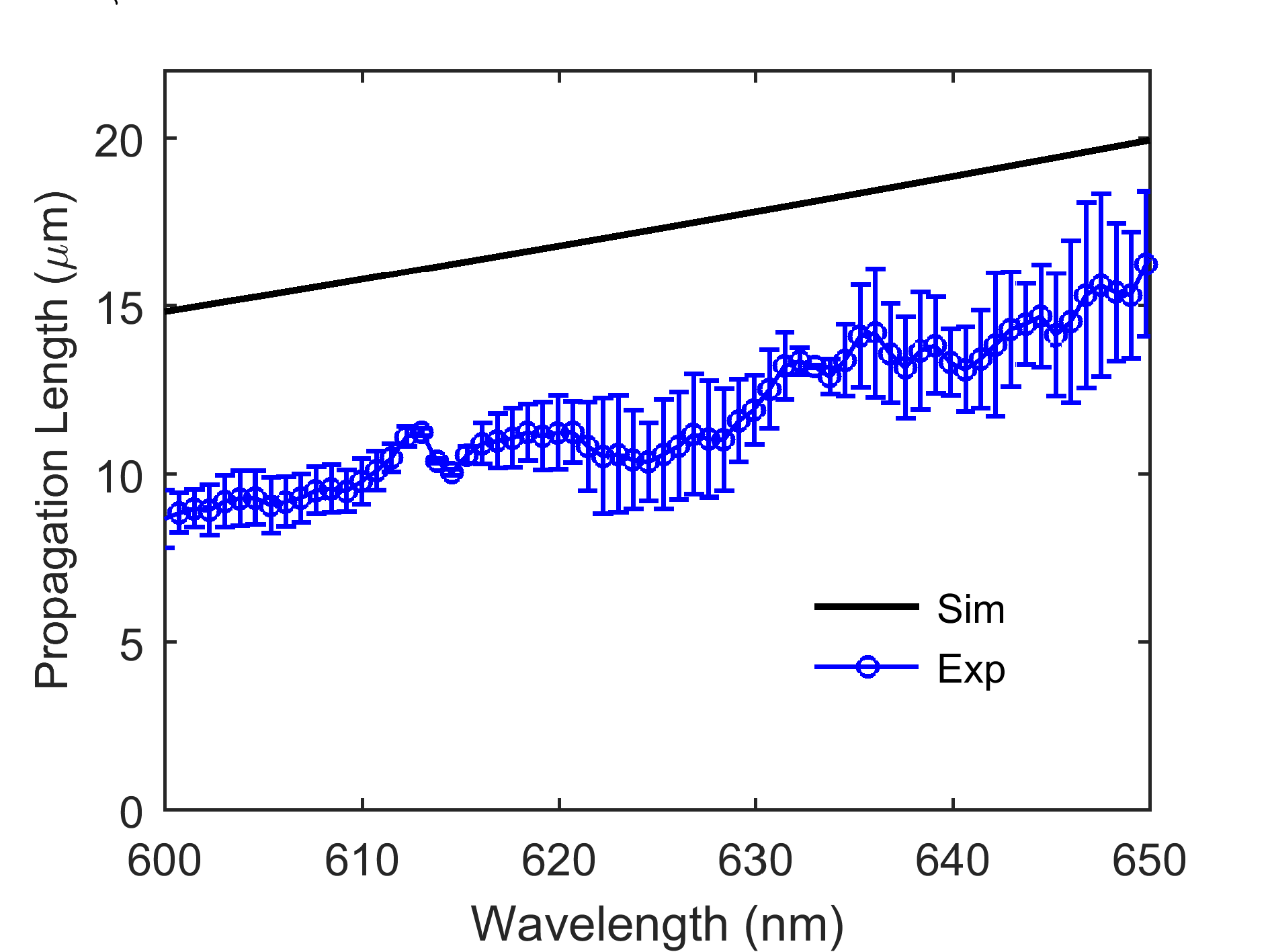


**Figure S5** Calculated and measured propagation length *L*_p_ of the configuration consisting of a 35-nm-thick SiO_2_ film on top of the Ag substrate. The experimentally measured SPP propagation length *L*_p_ closely matches the theoretical SPP propagation length.

**Section S7: Calculated coupling efficiencies of the decoupling gratings.**


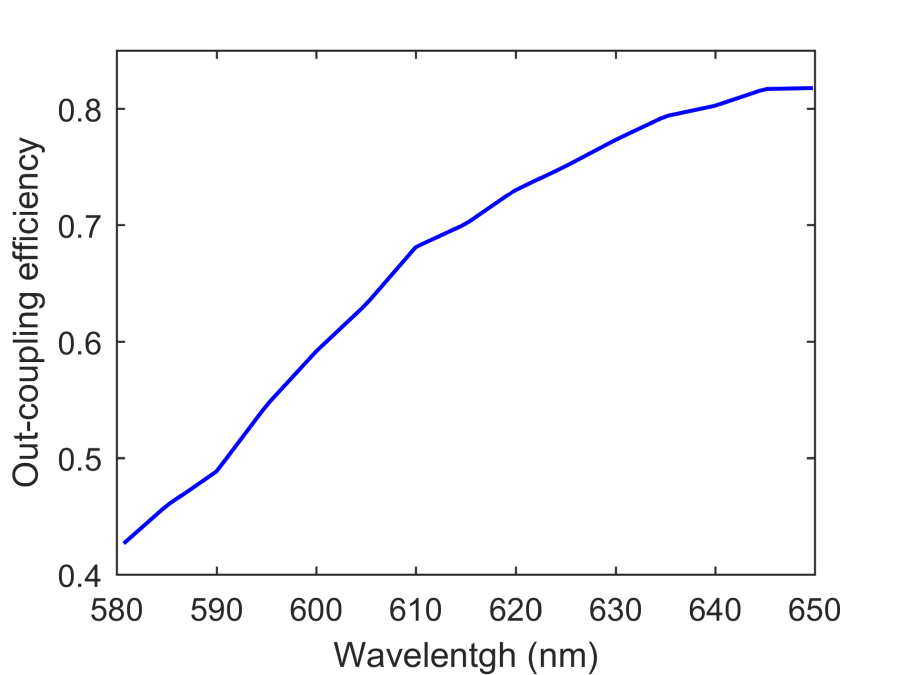


**Figure S6** Calculated coupling efficiencies of the decoupling gratings.

**Section S8: SPP excitation efficiencies versus the position of a scanned laser beam at *λ* = 610 nm and 650 nm.**


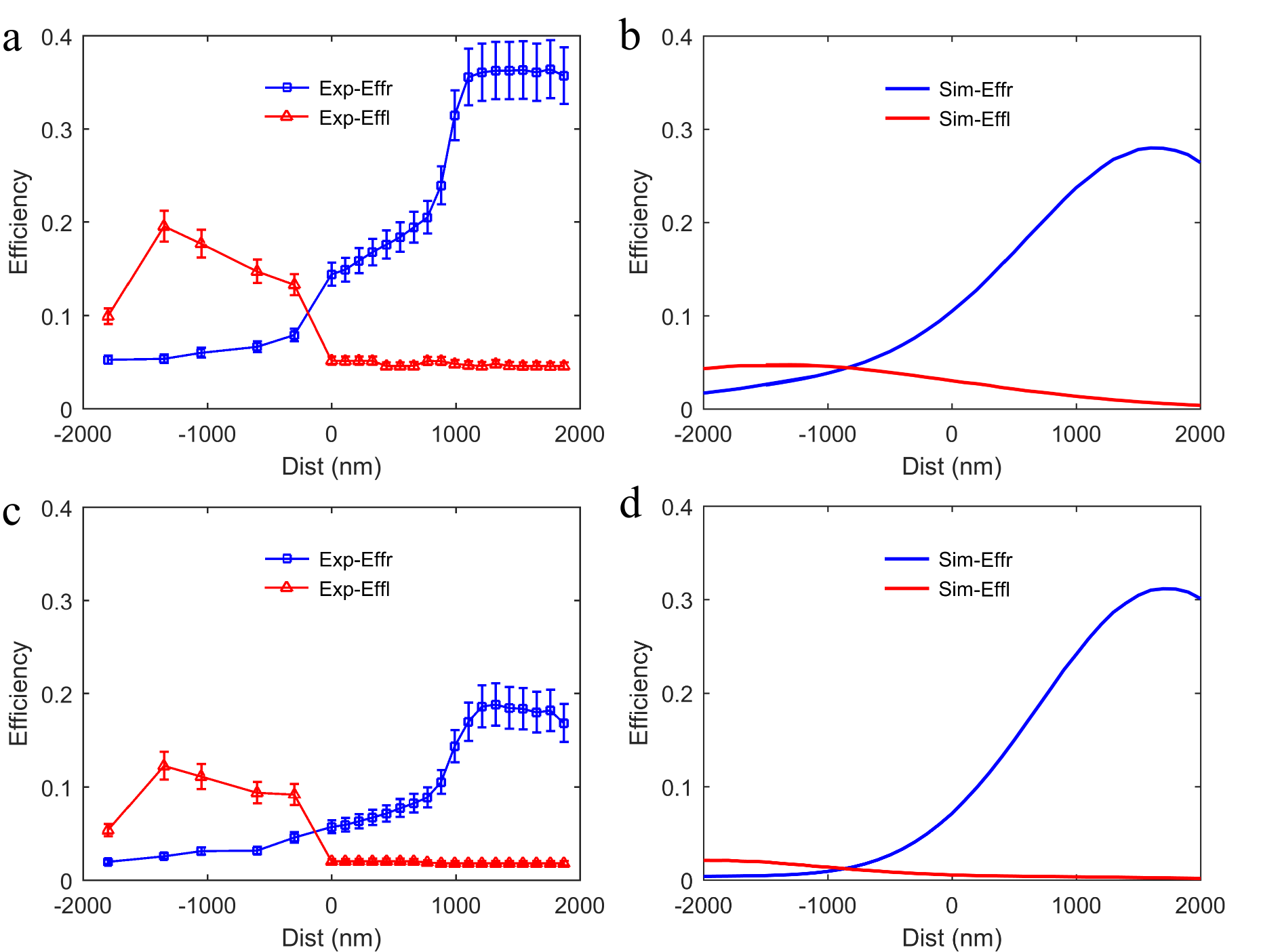


**Figure S7** Measured (a, c) and calculated (b, d) SPP excitation efficiencies versus the position of a scanned laser beam at *λ* = 610 nm (a, b) and 650 nm (c, d), respectively.

**Section S9: SPP excitation efficiencies versus the position of a scanned laser beam at *λ* = 633 nm for a regular grating coupler.**


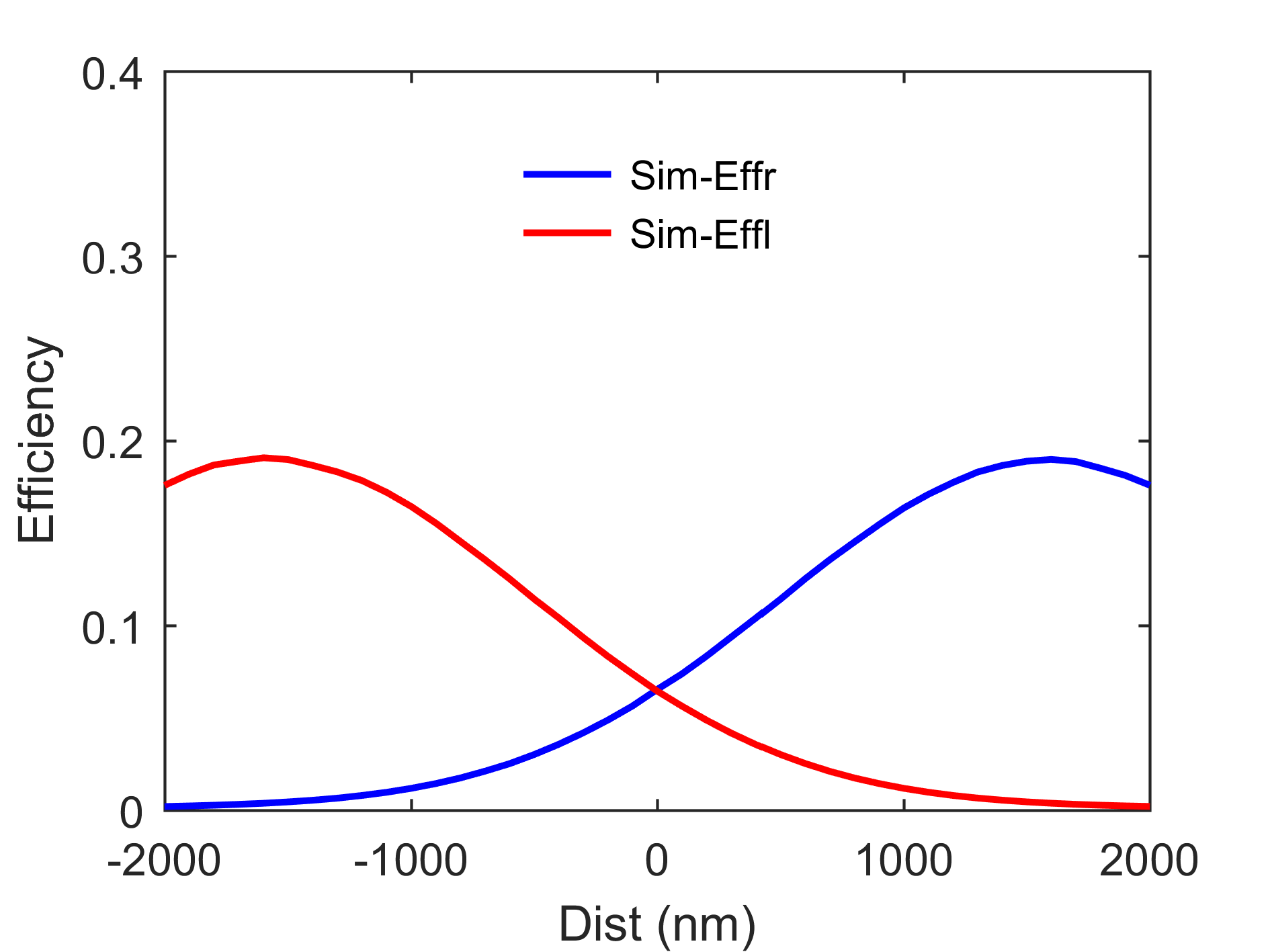


**Figure S8** Calculated SPP excitation efficiencies versus the position of a scanned laser beam at *λ* = 633 for a regular grating coupler, which consists of eight Ag nanobricks on top of a 35-nm-thick SiO_2_ and Ag substrate, with the center-to-center distance of *p* = 570 nm. The width and length of the brick (i.e. the element 4 in Figure 1b) are 82 nm and 148 nm, respectively.

**Section S10: Calculated and measured diffraction efficiencies of the periodic supercells for *x*-polarization.**


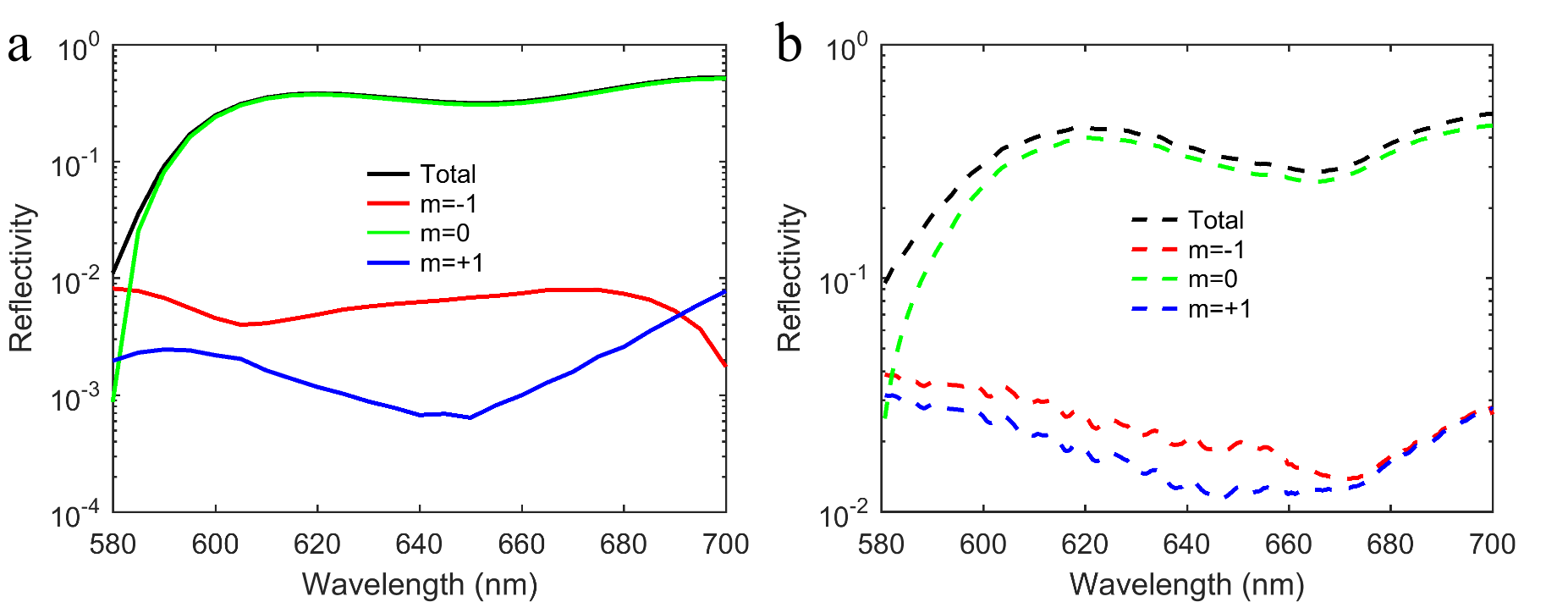


**Figure S9** Calculated (a) and measured (b) diffraction efficiencies of the metasurface illuminated with the *x*-polarized light in the directions of diffraction orders designed for the *y*-polarized incidence.

**Section S11: Calculated angular dependence of the SPP excitation for the *x*-polarization**

**
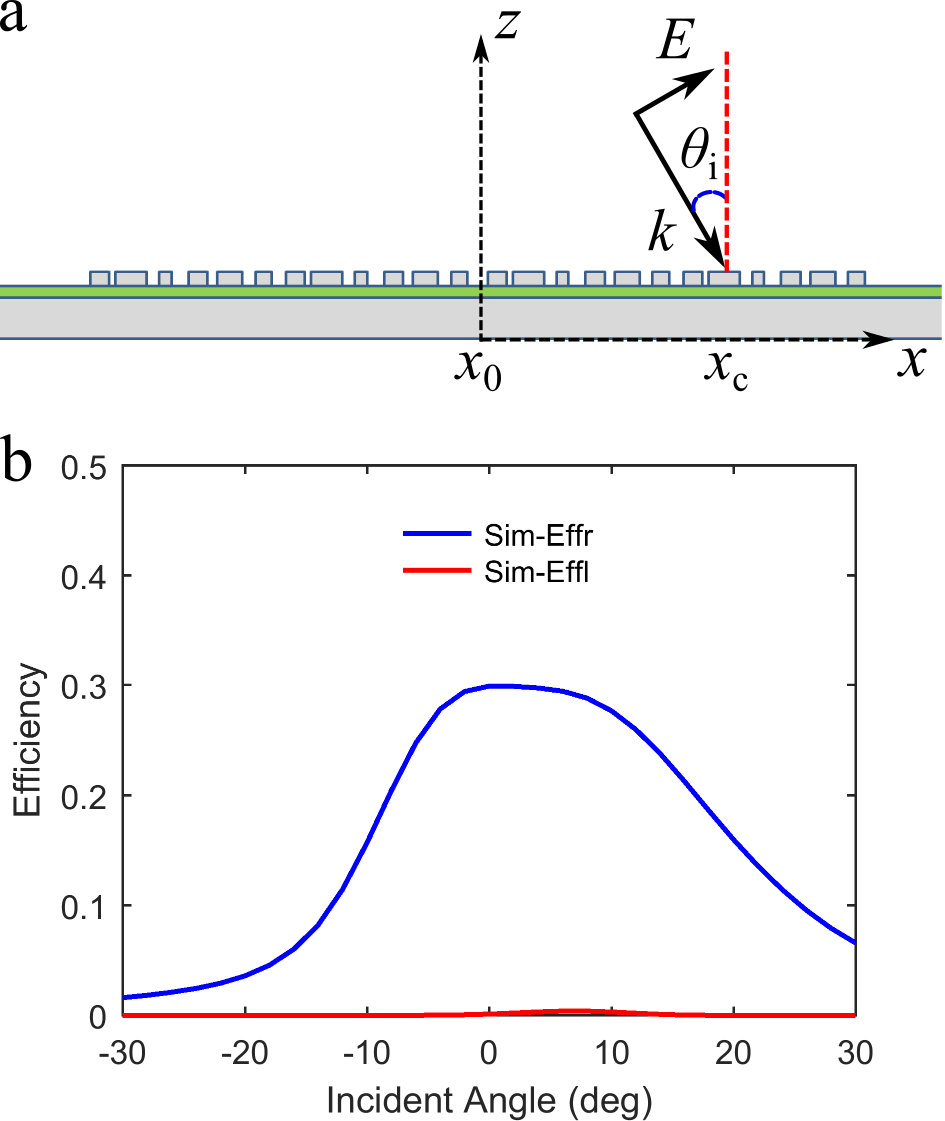
**

**Figure S10** Calculated angular dependence of SPP excitation efficiencies for the *x*-polarization at *λ* = 633 nm. (a) Side view of the simulated configuration. An *x*-polarized Gaussian beam (*w*_0_ = 2 μm) is obliquely incident on the center of the metasurface with an incident angle of *θ*_i_. The offset of Gaussian beam from the center of the SPP coupler is *Dist* = *x*_c_ – *x*_0_ = 1.71 μm. (b) The calculated SPP excitation efficiencies at different angles. When the *x*-component of the incident wave vector is pointing to the +*x*-axis, *θ*_i_ is set to be positive; otherwise, *θ*_i_ is negative. Unidirectional SPP excitation is sustained over a wide angle range, which is ascribed to the robust and flexible bridging between PW and SPPs since the momentum mismatch is compensated by the reflection phase gradient.^3,4^

**S12: Calculated and measured angular dependence of beam steering for the *y*-polarization**

When the incident angle *θ*_i_ is negative, the diffraction efficiencies remain constant up to a −30° angle of incidence as shown in Figure S11, which is due to the fact that the reflection phase gradient can be assumed to stay constant when *θ*_i_ is small. However, the measured zero-order diffraction is somewhat larger compared to the calculated value. When *θ*_i_ is positive and increasing gradually, the +1 diffraction order dominates at the beginning and then drops down quickly, becoming evanescent wave.

**
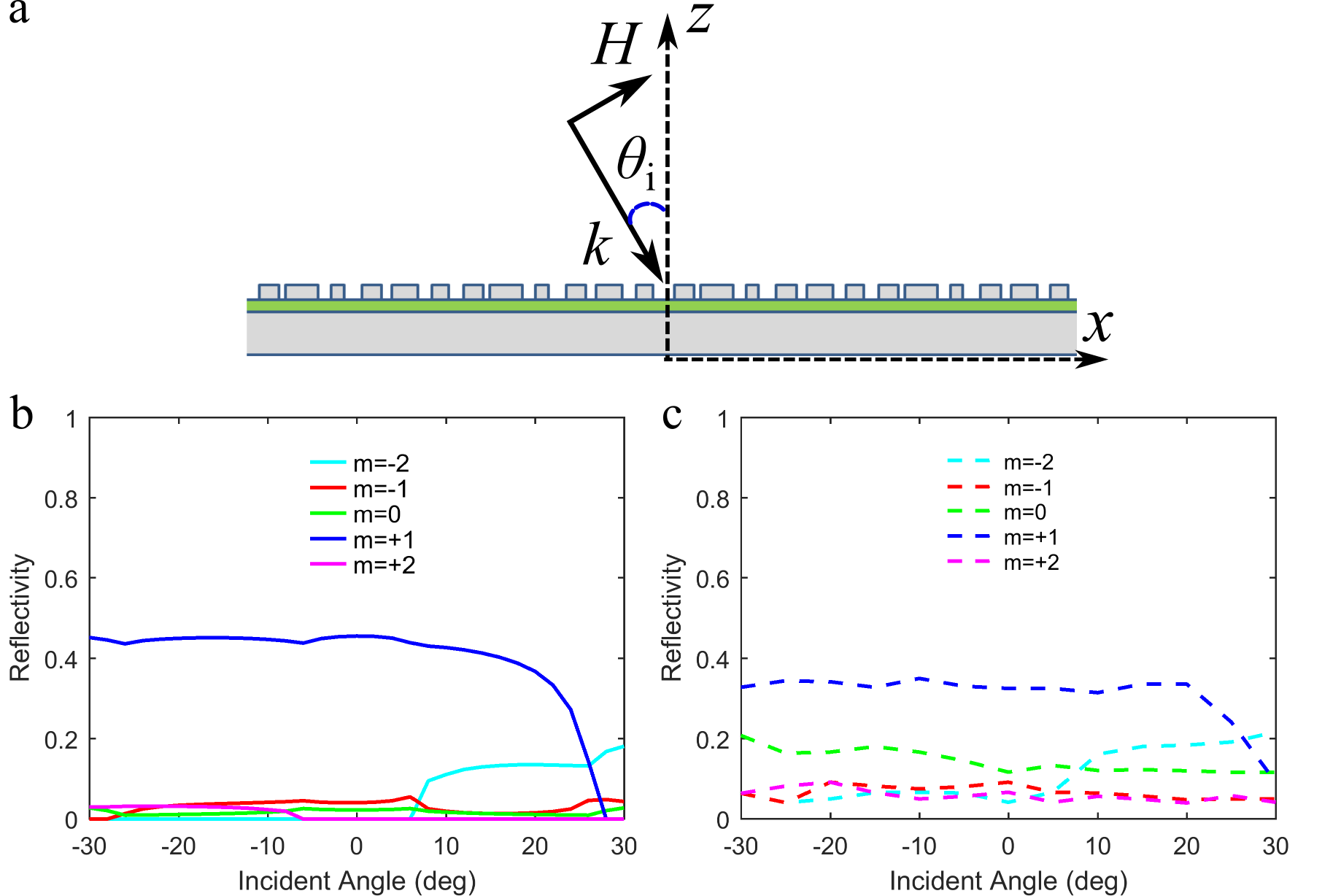
**

**Figure S11** Calculated and measured angular dependence of beam steering for the *y*-polarization at *λ* = 633 nm. (a) Side view of the simulation configuration. (b,c) The calculated (b) and measured (c) diffraction efficiencies at different angles. When the *x*-component of incident wave vector is pointing to the +*x*-axis, *θ*_i_ is set to be positive; otherwise, *θ*_i_ is negative.

**References**

1. Johnson PB, Christy RW. Optical Constants of the Noble Metals. *Phys Rev B* 1972; **6**: 4370-4379.
2. Pors A, Albrektsen O, Radko IP, Bozhevolnyi SI. Gap plasmon-based metasurfaces for total control of reflected light. *Sci Rep* 2013; **3**: 2155.
3. Sun S, He Q, Xiao S, Xu Q, Li X *et al*. Gradient-index meta-surfaces as a bridge linking propagating waves and surface waves. *Nat Mater* 2012; **11**: 426–430.
4. Pors A, Nielsen MG, Bernardin T, Weeber JC, Bozhevolnyi SI. Efficient unidirectional polarization-controlled excitation of surface plasmon polaritons. *Light Sci Appl* 2014; **3**: e197.
